# Adherence enhancing interventions for pharmacological and oxygen therapy in patients with COPD: protocol for a systematic review and component network meta-analyses

**DOI:** 10.1186/s13643-023-02326-x

**Published:** 2023-09-08

**Authors:** Omar Ammous, Stefan Andreas, Tim Friede, Regina Kampo, Sarah Schwarz, Maximilian Wollsching-Strobel, Susanna Salem, Wolfram Windisch, Tim Mathes

**Affiliations:** 1 Department of Medical Statistics, University Medical Center Göttingen, Göttingen, Germany; 2 Clinic for Pneumology/Krs. Kassel, Immenhausen, Germany; 3 Clinic for Cardiology and Pneumology, University Medical Center Göttingen, Göttingen, Germany; 4 Cologne Merheim Hospital, Department of Pneumology, Kliniken der Stadt Köln gGmbH, Cologne, Germany; 5 Witten/Herdecke University, Witten, Germany; 6 Department of Evidence-Based Health Services Research (Institute for Research in Operative Medicine), Witten/Herdecke University, Witten, Germany

**Keywords:** COPD, Adherence, Complex intervention, Risk of bias, GRADE, Network meta-analysis

## Abstract

**Background:**

Chronic obstructive pulmonary disease (COPD) is characterised by hyperinflation and expiratory airflow limitation due to long-term exposure to irritants. The variety and complexity of COPD treatment and the possible added comorbidities may make the patients find it difficult to cope with the required medications. That is why supporting patients’ adherence is critical because not taking medications correctly increases the risk of complications and creates an additional financial burden. A range of interventions aiming to improve patient adherence were used, and most of them are complex since they involve a mix of elements. Furthermore, despite the variety of available tools, assessing adherence is challenging because clinicians usually do not get a concrete judgement if their patients followed their treatment plan reliably. We aim to evaluate the effectiveness of adherence-enhancing interventions for COPD patients, explore which intervention (component) works for which patients and check the factors influencing the implementation and participant responses.

**Methods:**

We will perform a comprehensive literature search (Medline, Embase, Cochrane Library, trial registries) without restrictions on language and publication status, and we will include all controlled studies investigating the effect of adherence-enhancing intervention on patients with COPD. We plan to involve COPD patients in the systematic review development through two patient interviews (one before and one after the systematic review). Two reviewers will perform the screening, data extraction and risk of bias (ROB) assessment. For ROB, we will use ROB 2.0 to assess randomised controlled trials, and ROBINS-I to assess non-randomised studies. We will perform pair-wise random-effects meta-analyses and component network meta-analyses to identify the most effective components and combinations of components. We will use the Grading of Recommendations, Assessment, Development and Evaluation (GRADE) approach to assess the quality of evidence. To determine the degree of complexity, we will use the iCAT_SR checklist, and then, following a logical model, we will group the interventions according to prespecified criteria.

**Discussion:**

This systematic review aims to point out the most effective and implementable adherence-enhancing interventions by using methods for synthesising evidence on complex interventions and involving COPD patients all along with the review process.

**Systematic review registration:**

PROSPERO CRD42022353977

**Supplementary Information:**

The online version contains supplementary material available at 10.1186/s13643-023-02326-x.

## Background

Chronic obstructive pulmonary disease (COPD) is a common, preventable, and treatable disease characterised by hyperinflation and expiratory airflow limitation (a post-bronchodilator FEV1/FVC ratio of < 0.70) [1, 2]. It is a growing public health issue while becoming a leading cause of morbidity, mortality and hence requiring a high cost of healthcare [2, 3]. The disease appears after long-term inhalation of lung irritants like tobacco, fumes and professional exposure. It is associated with a decline in patients’ quality of life, physical capacity, social behaviour, and sleeping which may lead to severe disabilities [4, 5]. This is caused by the disease itself and the high number of comorbidities in COPD patients, making it the fifth leading cause of Disability-Adjusted Life Years lost in 2013 worldwide [2].

The prevalence of COPD increases continuously due to extended exposure to tobacco and the ageing global population [2]. A systematic review conducted in 2019 estimated the pooled prevalence of COPD to be 15.7% in men and 9.93% in women. It currently ranks third on the WHO list of the most deadly diseases worldwide, responsible for 3.23 million deaths in 2019 [6]. Numbers for prevalence, morbidity and mortality vary across countries. The highest prevalence of COPD among the WHO regions’ was reported in the Americas, while the lowest was in South-East Asia and Western Pacific regions [7]. In Germany, the Federal Environmental Agency estimates that about 12% of adults older than 40 years suffer from COPD. Furthermore, an increase from 6.8 million to 7.9 million COPD cases is expected by 2030 [8]. COPD is observed mainly in patients older than 70 years, with a greater pooled prevalence in men (15.7%) compared to women (9.93%) [6, 7].

COPD is also linked to a heavy financial burden due to health management costs, social expenses, and professional disability. About 56% (38.6 billion Euros) of the costs of treating respiratory diseases per year in the European Union are used to treat COPD [2]. These numbers are underestimated because they only include the direct health care cost and do not consider the costs for home-based care provided by family members and friends. Germany is the leading European country in terms of the annual per-patient cost of work productivity loss (5735 €) and is ranked third in the direct cost estimated per patient per year (7847 €) [3]. The management of COPD seems to be the most expensive [3]. Indeed, managing COPD involves a set of interventions according to the state and the stage of the disease, which is mainly based on bronchodilators, corticosteroids and antibiotics. Other interventions include pulmonary rehabilitation, oxygen therapy, and ventilatory support are considered for advanced/acute stages [2]. The principal aim of COPD interventions is to sustain a good quality of life, physical activity, and avoid exacerbations. That is why COPD patients must adhere to their treatment and take their medications conveniently. Adherence to COPD medication reduces hospitalisation risk, complications, mortality, and costs [9]. On the other side, non-adherence is associated with multiple adverse outcomes. It is the primary reason behind increased exacerbation risk and treatment failure [10–12].

Medication/device adherence can be defined as the extent to which a patient’s behaviour corresponds with the prescribed therapy or therapy regime—including time, dosing/intensity and interval of intake/application [13]. The WHO defined it as “the extent to which the patient follows medical instructions…” [14]. According to the WHO, around half of patients with chronic conditions do not adhere to their treatments [14–16]. Studies estimated that more than half of COPD patients could be considered non-adherent to their therapy [10, 17]. Factors that might negatively impact adherence are socioeconomic status and high age [7]. Both factors are prevalent in COPD patients [8]. Unlike clinical trials, where adherence is up to 90% [18–21], adherence in clinical practice ranges between 10 and 40% [18, 22–24]. The variety and complexity of COPD treatment and the possible added comorbidities may make the patients overwhelmed and unable to cope with the required medications. Therefore, supporting patients’ adherence is critical because not taking medications effectively and correctly increases the risk of complications and creates an additional financial burden.

Moreover, many factors surround adherence, and it is difficult to determine which are the most impactful [25]. Some of these factors are related to the patient itself, like their beliefs, perceptions, understating of the disease, cognitive status, expectations, and the presence of comorbidities. Depression and anxiety, for example, can be an obstacle to adherence. It has been reported that depressed COPD patients are three times more prone to non-adherence [26, 27]. Moreover, some studies addressed the possible opposite relationship between adherence and health-related quality of life (HRQoL) [11, 28, 29]. Although adherence can improve HRQoL by increasing treatment effectiveness, good life quality can generate non-adherence. Other factors may also impact compliance. For instance, physicians in their chosen management strategy and in dealing with their patients’ overall context, including familial and socioeconomic status (e.g. caregivers, illiteracy, unemployment and poverty) [30].

The process of adherence consists of three components: initiation, implementation, and discontinuation [13]. Initiation is the date of the first dose taken, and then the process continues with implementation, which is the extent to which a patient copes with his prescribed medications. It also corresponds to the time from initiation until the last dose taken, also known as persistence. Discontinuation happens when the patient stops taking his subsequent required treatment. A range of interventions aiming to improve patient’s adherence were used, and most of them are complex since they involve a mix of elements (i.e. health education, behavioural and cognitive therapies, psychosocial support, and devices). Some tools used to assess adherence are questionnaires and self-reported methods, like the Battala test, Morisky Green test, inhaler adherence scale and Haynes and Sackett method [18, 30, 31]. Other reported approaches are electronic monitors, canister weighing, analysis of pharmacy records, and the ratio of doses taken/doses prescribed [30]. Despite the variety of measuring adherence, no gold standard exists and no method is flawless [30, 32]. Also, assessing adherence is challenging because clinicians usually don’t get a concrete judgement if their patients followed their treatment plan correctly. Usually, the issue of non-adherence rises with the non-improvement or decline in the patient’s state of health without other possible causes. Therefore, it is essential to undertake a more specific approach and consider intervention complexity when exploring factors surrounding adherence success.

Many of the previous systematic reviews on adherence have been inconclusive, particularly because of heterogeneous results (e.g. [33]). This will be one of the first systematic review that uses a broad spectrum from the toolbox of methods for systematic reviews of complex interventions to synthesise evidence on adherence-enhancing interventions. Indeed, it will provide essential insights into the value (e.g. explanation of heterogonous findings) and limitations of evidence synthesis methods for complex interventions for analysing adherence interventions.

## Objectives

The aim of this systematic review is to evaluate the effectiveness of adherence-enhancing interventions, alone or compared to other adherence-enhancing interventions, for patients with COPD. In addition, we will explore which intervention (component) works for which patients and under which circumstances, using methods for synthesising evidence on complex interventions.

## Methods and analyses

This protocol was registered with the International Prospective Register of Systematic Reviews (PROSPERO) on 29 August 2022 with registration number CRD42022353977 and has been written under the Preferred Reporting Items for Systematic Review and Meta-analyses Protocols (PRISMA-P) guidelines (see checklist in the Additional file 1) [34]. We will perform the review according to the Cochrane handbook for systematic reviews of interventions [35].

### Type of studies

We will include randomised controlled trials (RCT), cluster-randomised controlled trials, non-randomised controlled trials, cohort studies with a concurrent comparison group and controlled-before-after studies and interrupted-time series studies [36]. The latter will be included if three measures before and after the intervention were conducted [37].

### Type of participants

Patients with COPD diagnosed according to international standards [2]. We will include all studies, in which at least 80% are COPD patients or that report the results of COPD patients and other patients (e.g. asthmatic patients) separately.

### Type of interventions

We will include studies that analyse an intervention which could have a direct or indirect positive impact on patient adherence. We are particularly interested in studies that aim to improve the management, intake or administration of the entire COPD pharmacological and oxygen COPD therapy (i.e. therapy management programs) because we assume that adherence problems, at least in part, arise from the complexity of the whole COPD therapy. Moreover, the optimal treatment outcome can only be reached if all different types of therapies are correctly used and are geared to each other. Nevertheless, we will include studies that examined adherence interventions only targeting certain types of therapies (e.g. inhalers) as some patients might only get a single type of therapy. However, we will include inhalation technique training interventions only if they were part of an adherence intervention. In this way, we will receive additional evidence on the effectiveness of individual intervention components (see section Data synthesis).

All types of adherence measures are eligible. This includes education (e.g. information material), behavioral counselling (e.g. motivational interviewing), managing support, reminders, and incentives.

### Type of comparators

The comparator study arm must be either no adherence-enhancing intervention (i.e. usual care) or another adherence-enhancing intervention.

### Type of outcome measures and prioritisation

We plan to perform two patient interviews (one before and one after the systematic review) and follow a sequential approach to integrating qualitative and quantitative information. The first interview aims to understand patients’ needs and prioritise the selection of the outcomes. The second interview will be conducted after the evidence synthesis to present the results to patients.

Overall, the outcomes may be as follows:

#### Primary outcomes

- Adherence: it is categorised into three stages: initiation, implementation and discontinuation. Sometimes, the term ‘persistence’ is added. We will analyse each component as a part following what is available in the literature.
- COPD Exacerbations: defined as an increase in dyspnoea and/or cough and sputum that worsens in less than 14 days [2].
- Functional exercise capacity: without restrictions on scales.
- Health-related quality of life (HRQoL): without restrictions on scales.

#### Secondary outcomes

- Hospital admission: if possible, we will analyse hospitalisation beyond the emergency department (e.g. pneumology department, intensive care unit) separately.
- Mortality
- Inhaler technique
- Respiratory function:
  - Forced expiratory volume at 1 s (FEV1)
  - Tiffeneau coefficient: FEV1/FVC
- Adverse events

### Search methods for identification of studies

#### Electronic searches

We will search for all published and unpublished studies regarding adherence-enhancing interventions for COPD. We will develop a comprehensive literature search strategy in collaboration with an experienced librarian and without restrictions on language and publication status (e.g. published, unpublished, ongoing). The search strategy will follow the Peer Review of Electronic Search Strategies (PRESS) guideline.

We will search the following databases to identify relevant studies:

- MEDLINE and MEDLINE in process (via PubMed): inception to present;
- EMBASE (via EMBASE): inception to present;
- CENTRAL (via Cochrane Library): inception to present;
- CINHAL (via EBSCO): inception to present;

We will search manually for additional studies by:

- forward and backward reference screening of all included primary studies;
- forward and backward reference screening of relevant and related systematic reviews.

We will search the following trial registries:

- ClinicalTrials.gov
- German Clinical Study Register (DRKS)
- International Clinical Trials Registry Platform (ICTRP)

The search strategy will be combined with a highly sensitive filter for RCTs and a study filter for comparative non-randomised studies [38, 39]. Our PubMed search strategy is detailed in Table 1.

**Table 1:** PubMed search strategy

**PubMed (16.09.2022; 2609 Hits)**
(“chronic obstructive lung disease”[tiab] OR “chronic obstructive lung diseases”[tiab] OR “chronic airflow obstruction”[tiab] OR “chronic airflow obstructions”[tiab] OR “chronic airway obstruction”[tiab] OR “chronic airway obstructions”[tiab] OR “chronic obstructive lung disorder”[tiab] OR “chronic obstructive lung disorders”[tiab] OR “chronic obstructive pulmonary disease”[tiab] OR “chronic obstructive pulmonary diseases”[tiab] OR “chronic obstructive pulmonary disorder”[tiab] OR “chronic obstructive pulmonary disorders”[tiab] OR copd[tiab] OR coad[tiab] OR cobd[tiab] OR cold[tiab] OR emphysema[tiab] OR “Pulmonary Disease, Chronic Obstructive”[mh])
AND
(adherence[tiab] OR adherent[tiab] OR adhere[tiab] OR nonadherence[tiab] OR non-adherence[tiab] OR nonadherent[tiab] OR non-adherent[tiab] OR compliance[tiab] OR “patient compliance”[mh] OR medication adherence[mh] OR compliant[tiab] OR comply[tiab] OR noncompliance[tiab] OR non-compliance[tiab] OR noncompliant[tiab] OR non-compliant[tiab] OR “patient compliance”[mh])
AND
(((cohort[all] OR (control[all] AND study[all]) OR (control[tw] AND group*[tw]) OR epidemiologic studies[mh] OR program[tw] OR clinical trial[pt] OR comparative stud*[all] OR evaluation studies[all] OR statistics as topic[mh] OR survey*[tw] OR follow-up*[all] OR time factors[all] OR ci[tw]) NOT ((animals[mh:noexp] NOT humans[mh:noexp]) OR comment[pt] OR editorial[pt] OR review[pt] OR meta analysis[pt] OR case report[tw] OR consensus[mh] OR guideline[pt] OR history[sh]))
OR
(“Randomized controlled trial”[pt] OR “controlled clinical trial”[pt] OR random* [tiab] OR “clinical trials as topic”[mh:noexp] OR trial[ti] NOT ((animals[mh:noexp] NOT humans[mh:noexp]) OR comment[pt] OR editorial[pt] OR review[pt] OR meta analysis[pt] OR case report[tw] OR consensus[mh] OR guideline[pt] OR history[sh])))

#### Searching other resources

We will check the reference lists of all included primary studies and systematic reviews on the same topic for additional references. We will search EPISTEMONIKOS to identify relevant systematic reviews. When appropriate, we will contact experts in the field to ask for any ongoing trials or newly published papers.

### Data collection and analysis

We will follow the recommendations of the Cochrane handbook when conducting the screening process, data extraction and management [35].

### Selection of studies

Two review authors, one with clinical expertise and one with methodological expertise will independently perform study selection. They will screen the titles and abstracts of the search results using Rayyan and code them as ‘retrieve’ (eligible or potentially eligible/unclear) or ‘do not retrieve’. Subsequently, the same reviewers will retrieve the full text of all potentially relevant titles/abstracts and screen them for inclusion while recording the reasons for excluding ineligible studies. In case of discrepancies, a discussion will determine eligibility until consensus. If necessary, a third person will be involved. We will record the selection process in sufficient detail to complete a PRISMA flow diagram [34].

### Data extraction and management

Two review authors will use a data collection form piloted on at least one study in the review to extract characteristics from included studies. One of the reviewers will be a statistician or epidemiologist. We will extract data using an Excel spreadsheet. Any missing information will be recorded as unclear or not described. Descriptive data (e.g. study characteristics) will be extracted by one reviewer and verified by a second. Two review authors will independently extract outcome data from included studies [40]. We will report if outcome data were not reported in a usable way. If multiple reports from the same study are identified, we will directly extract data from all reports into a single data collection form.

We will extract the following study characteristics from the included studies:

- General information: study ID, author contact details, study centres, locations, and setting
- Methods: study design, total study duration.
- Participants: inclusion criteria, exclusion criteria, total number randomised, number randomised per group, age, gender, COPD stage, smoking history, clusters (if applicable)
- Intervention/comparison groups: we will follow the template for intervention description and replication (TIDieR) [41]. For further details, please refer to Table 2.
- Outcomes: primary and secondary outcomes, baseline measures, measurement instrument, and time points.
- Notes: funding for studies and notable conflicts of interest of trial authors.

**Table 2:** The TIDieR (Template for Intervention Description and Replication) Checklist [41]

**Item**	**Item number**	**Extraction**
**Brief name**	**1**	Extract the name or a phrase that describes the intervention.
**Why**	**2**	Extract any rationale, theory, or goal of the elements essential to the intervention.
**What**	**3**	Materials: extract any physical or informational materials used in the intervention, including those provided to participants or used in intervention delivery or in training of intervention providers. Provide information on where the materials can be accessed (e.g. online appendix, URL).
	**4**	Procedures: extract each of the procedures, activities, and/or processes used in the intervention, including any enabling or support activities.
**Who provided**	**5**	For each category of intervention provider (e.g. psychologist, nursing assistant), extract their expertise, background and any specific training given.
**How**	**6**	Extract the modes of delivery (e.g. face-to-face or by some other mechanism, such as internet or telephone) of the intervention and whether it was provided individually or in a group.
**Where**	**7**	Extract the type(s) of location(s) where the intervention occurred, including any necessary infrastructure or relevant features.
**When and how much**	**8**	Extract the number of times the intervention was delivered and over what period of time including the number of sessions, their schedule, and their duration, intensity or dose.
**Tailoring**	**9**	If the intervention was planned to be personalised, titrated or adapted, then extract what, why, when, and how.
**Modification**	**10**	If the intervention was modified during the course of the study, extract the changes (what, why, when, and how).
**How well**	**11**	Planned: if intervention adherence or fidelity was assessed, extract how and by whom, and if any strategies were used to maintain or improve fidelity, extract them.
	**12**	Actual: if intervention adherence or fidelity was assessed, extract the extent to which the intervention was delivered as planned.

### Risk of bias assessment

Two review authors will independently assess the risk of bias (RoB) of each outcome following criteria outlined in the Cochrane Handbook for Systematic Reviews of Interventions [35]. Discrepancies will be resolved in a discussion until a consensus is reached.

The risk of bias of RCTs will be assessed with the Cochrane risk of bias 2.0 tool [42]. We will use the RoB 2 Excel tool to complete RoB 2 assessment, and the robvis tool to generate “traffic light” plots of the domain-level judgements for each outcome and weighted bar plots of the distribution of ‘Risk of bias’ judgments within each bias domain [43]. We will assess the risk of bias, which can be low, some concern or high, according to the following domains:

- Bias arising from the randomisation process
- Bias due to deviations from intended interventions
- Bias due to missing outcome data
- Bias in measurement of the outcome
- Bias in selection of the reported result

Our effect of interest is starting intervention. We will judge each outcome as being at low risk, some concerns, or high risk according to the RoB2 algorithm. We will provide a quote from the study report and a justification for our judgment in the ‘Risk of bias’ table. We will report information on the risk of bias relates to unpublished data or correspondence with a trialist.

For cluster-RCTs, we will use the test version of RoB 2.0 for this study design (10 November 2020, revised 18 March 2021) [44].

The risk of bias of non-randomised studies will be assessed with ROBINS-I [35, 45], considering it low, moderate, serious or critical. We will assess the following domains:

- Bias due to confounding
- Bias in selection of participants into the study
- Bias in classification of interventions
- Bias due to deviations from intended interventions
- Bias due to missing data
- Bias in measurement of the outcome
- Bias in selection of the reported results

For baseline confounding factors, we consider age, socioeconomic status (e.g. income, education), hospitalisation and COPD exacerbation in the last 6 months. For time-varying confounding factors, we consider COPD severity.

We will report the risk of bias assessment in the Results section. It will also be part of the GRADE assessment of the certainty of evidence (along with precision, directness, consistency, and publication bias). When considering treatment effects, we will take into account the risk of bias for the studies that contribute to that outcome. Our primary analysis will include all studies without considering the risk of bias assessment.

In addition to risk of bias, we will assess the quality of recruitment strategy [46].

### Measures of treatment effect

The choice of the summary effect depends on the type of the outcome and how they were reported. We will use risk ratio (RR) for dichotomous outcomes, and we will analyse continuous outcomes as a mean difference (MD) or, when needed, as a standardised mean difference (SMD) (i.e. combine different scales).

For time-to-event data (as reported by the authors), our treatment effect will be the hazard ratio (HR).

### Data synthesis

To determine the degree of complexity, we will use the iCAT_SR checklist [47]. Based on this assessment and the logical model, the findings will be systematically tabulated and graphically displayed [48].

We will group the interventions according to the following criteria to explore which factors might affect the effectiveness of adherence-enhancing interventions:

- Intervention target: inhalers, oral medications, oxygenation, or multiple types of adherence intervention: education, behavioural counselling, managing support, reminder and incentives
- Number of effective components (e.g. education [1] vs. education plus reminder [2], as determined with iCAT_SR))
- Behaviours or actions of intervention recipients or participants to which the intervention is directed (as determined with iCAT_SR)
- The duration of the intervention (short, medium, and long-term intervention)
- Degree of tailoring (as determined with iCAT_SR) to the individual patients
- Targeted adherence type: unintentional vs. intentional non-adherence [49]
- Optionally, further if suggested by the patient interviews or logical model.

We will prepare harvest plots and forest plots (with or without pooled estimates) [50] and perform meta-analyses for studies with sufficient clinical and methodological homogeneity. Then, statistical heterogeneity will be explored using prediction intervals.

We will perform two types of meta-analyses to assess the effectiveness of adherence-enhancing interventions (components). We will run pair-wise random-effects meta-analyses using the Paule-Mandel heterogeneity variance estimator and (modified) Hartung-Knapp confidence intervals (CIs) to determine the overall effectiveness of the adherence interventions [51]. The variance correction factor for the Hartung-Knapp confidence intervals will only be applied if the 95%-CIs of the conventional Hartung-Knapp CIs are narrower than Wald-type CIs. We will use beta-binomial models for meta-analyses of less than five studies and zero event studies [52, 53], Bayesian random-effects meta-analyses with weakly informative priors for tau-square for sensitivity analysis, and Bayesian random-effects meta-analyses with weakly informative priors for the treatment effect for zero events studies [54, 55].

In addition, we will conduct random-effects component network meta-analyses to identify the most effective components and combinations of components [50, 56]. We will check if the transitivity assumption is met. Network meta-analyses has recently been shown promising to offer additional insights when analysing adherence interventions [57]. In these models, each adherence intervention component (e.g. education or reminder) will be treated as a separate component (separate node in the network). We will build two types of models. First, an additive model assuming that each component has a fixed effect. This model will answer the question of the most effective adherence intervention/component. The second is an interaction model in which different types of adherence interventions can interact. This model will answer the question of which adherence-enhancing components have the strongest synergies.

All analyses will be performed using R (package meta, bayesmeta, and netmeta) and SAS.

In addition to the meta-analyses, we will carry on a structured narrative synthesis to understand which patients benefit from which interventions and contexts. For this analysis, the results of the structured tabulations and graphical displays will be contrasted with the patient characteristics and study characteristics (e.g. setting). Furthermore, the narrative synthesis will incorporate information from the second round of patient interviews.

### Subgroup analysis

Furthermore, we plan to perform the following subgroup analysis:

- Gender
- Age (< 70 and ≥ 70 years)

### Sensitivity analysis

We plan to conduct a sensitivity analysis by removing RCTs at high risk of bias and some concern, and non-RCTs judged at critical and serious risk of bias.

### Confidence in cumulative evidence (GRADE assessment)

We will assess the certainty of the body of evidence for each prioritised outcome with GRADE and prepare a summary of findings tables [58]. We will use the methods and recommendations described in the Cochrane handbook [35] using GRADEpro GDT software [59]. We will justify all decisions to downgrade the quality of studies using footnotes and make comments to explain the summary of the evidence.

We will follow the GRADE guidance for using ROBINS-I to facilitate the integration of non-randomised and randomised studies in the body of evidence [60].

### Meta-bias

To detect reporting bias, we will compare the study protocol with the published report, if possible. We will use the ROB-ME tool to assess the risk of bias due to missing evidence in the synthesises [61]. We will try to contact the study authors to identify missing or partially reported data. If more than 10 studies are included in the meta-analysis, we will create a funnel plot to explore publication bias. We will use the Copas selection model-based tests suggested by Duan et al. to test for publication bias [62].

### Patient and public involvement

In light of the complexity of COPD treatment regimes, suitable strategies to increase adherence must be developed together with patients to ensure a successful implementation in routine care. To involve patients in the project, two patient interviews are planned; one before and one after the systematic review of evidence on adherence-enhancing interventions. The integration of this qualitative and quantitative information from the systematic review will follow a sequential approach [63]. That is, the review question is informed by qualitative data. Subsequently, quantitative data is collected and finally, the interpretation and conclusion of the quantitative data is informed by qualitative information.

The first interviews with COPD patients will be conducted before starting the systematic literature review. Focus group interviews will be performed using a semi-structured interview guideline. The qualitative content analysis will be carried out computer-assisted via MAXQDA according to the descriptions of Kuckartz and the Grounded Theory Methodology in accordance with the goal of the content analysis [64]. The results of this analysis will be used to condense the interview material into deductive-inductive main, and sub-categories, which can be integrated as weighting points for the systematic review. In these interviews, patient views on adherence (facilitators, barriers, own strategies for correct intake) and patient-relevant outcomes will be elaborated. The findings will be incorporated into a logical model to inform the research question (e.g. refinement of eligibility criteria), the synthesis, and the applicability assessment [65, 66].

## Discussion

Supporting patient adherence is of major importance, particularly for complex treatment strategies [67]. COPD treatment encompasses several therapeutic components, including device-based therapies such as long-term oxygen therapy and various drug treatments. This complex therapeutic regime requires a high level of information, education, and skills to handle the therapy correctly. Different interventions can support adherence, for example, video-assisted education courses, mobile apps, and physician feedback [68]. However, it is unclear which adherence-enhancing measures should be used in clinical practice. Most adherence-enhancing interventions fulfil the criteria constituting a complex intervention [48]. Therefore, this systematic review aims to evaluate the effectiveness of adherence-enhancing interventions for COPD patients in general and explore which intervention (components) work for which patients and under which circumstances, using methods for synthesising evidence on complex interventions [48, 69].

Due to the increasing burden of COPD and the increasing complexity of treatment, the scientific evaluation of adherence to specific COPD therapy has increased in recent years [70]. Although a Cochrane systematic review has already explored the topic [71], the main strength of our analyses will be that we explore possible heterogeneity using methods for analysing complex interventions and assess the applicability of the results to the German health care system. Based on the findings from the evidence synthesis and the interviews with patients, a multi-component, adherence-enhancing concept that could be tailored to the patient’s individual needs and that accounts for the specifics of the German context of COPD care will be developed. It is aimed to evaluate the developed program in a pragmatic cluster-randomised controlled trial in a subsequent study project. If the evidence-based adherence measures are implemented in practice, they might potentially reduce the treatment burden (e.g. adverse events, coping with medication complexity). Furthermore, as low adherence is associated with increased morbidity (e.g. hospitalisations), mortality and quality of life, an effective adherence intervention could improve these patient-relevant outcomes.

We will create a staged logic model following the recommendations and guidelines about the taxonomy of logic models [72], and Rohwer et al. [73] recommended strategy and design. We plan to create two types of logic models (system-based and process-oriented) since both may provide additional insight and description of the intervention. For the system-based logic model, we followed some of the design of a Cochrane review since it was a concrete example presented by the authors [74]. The logic models will be revised after the patient interview. Further details about the logic models are in the Additional files 2 and 3.

The second interview will be conducted after synthesising the evidence to present the results to patients. Here, qualitative content analysis will be used to assess the patients’ opinions structurally and to form new theories on successful adherence-enhancing measures for COPD patients. These interviews will focus on gathering information on factors that might hinder or facilitate the integration of the proposed adherence measures in the patients’ daily lives, to identify adherence measures that are most suitable for the individual patient. Thus, adherence measures may be more accepted and implemented by patients.

### Supplementary Information

**Additional file 1.** PRISMA-P 2015 Checklist.

**Additional file 2.** Process-oriented logic model.

**Additional file 3.** System-based logic model.

## Abbreviations

COPD: Chronic obstructive pulmonary disease
GRADE: The Grading of Recommendations Assessment, Development and Evaluation
HRQoL: Health-related quality of life
MD: Mean difference
RCT: Randomised clinical trial
RoB: Risk of bias
RR: Relative risk
SMD: Standardised mean difference
