# Supplementary material for: Adherence enhancing interventions for pharmacological and oxygen therapy in patients with COPD: protocol for a systematic review and component network meta-analyses
Source: Syst Rev. 2023 Sep 8;12:159. doi: 10.1186/s13643-023-02326-x (PMC10486002; doi:10.1186/s13643-023-02326-x)
Supplement: Supplementary file 2 — Additional file 2. Process-oriented logic model. [file 13643_2023_2326_MOESM2_ESM.docx]

Treatment adaptation

Pharmaceutical care

Devices – Health information technology

Adjustment of thoughts, feelings and behaviour

Reduction of negative thinking and maladaptive behavioural patterns

Detection and management of adherence barriers

Better COPD monitoring

**Short-term**

**Outcomes**

Patient involvement in healthcare decision making

Better acceptance/ understanding of COPD

Stimulate the will to change/ Breaking away from ambivalence

**Intermediate effects**

**Direct effets**

Patient education

Motivational therapy

**Intervention components**

**Adherence Enhancing Interventions**

Adaptation skills

Sollicitation of intrinsic motivation

Better acceptance of treatments

Cognitive behavioral therapy

Selfcare skills

Enhancement of adherence

**Long-term**

**Medium-term**

Fewer exacerbations and hospitalisations

Improved exercise capacity

Better life quality

Lower respiratory mortality
