# Supplementary material for: Adherence enhancing interventions for pharmacological and oxygen therapy in patients with COPD: protocol for a systematic review and component network meta-analyses
Source: Syst Rev. 2023 Sep 8;12:159. doi: 10.1186/s13643-023-02326-x (PMC10486002; doi:10.1186/s13643-023-02326-x)
Supplement: Supplementary file 3 — Additional file 3. System-based logic model. [file 13643_2023_2326_MOESM3_ESM.docx]

**Intervention theory:** enhance patients’ adherence by influencing different aspects surrounding COPD pharmacological and oxygen therapy compliance

**Execution**

- A couple of weeks to a year
- Sessions: once or several times per week or per month

**Intervention design**

**Components**

- Patient education
  - Understanding of COPD
  - Patients’ needs and expectations
  - Address barriers to medication adherence
  - Use of inhalers training
- Devices and health-information technology
- Pharmaceutical care and pharmacy-led interventions
- Adjustment, modification, revision, and variation in treatment protocols
- Cognitive behavioral therapy
- Motivational interviewing

**Outcomes**

**Intermediate outcomes**

*Process outcome:* overcoming adherence barriers (e.g. context)

*Behaviour outcomes:* adjustment of beliefs, attitudes and perceptions

*Surrogate outcomes:* spirometry assessments

**Health outcomes**

*Short-term*

- Enhancement of adherence
- Adequate inhalation technique

*Medium-term*

- Fewer exacerbations and hospitalisations

*Long-term*

- Improved exercise capacity
- Better life quality
- Lower respiratory mortality

**Population**

- Patients with COPD
- COPD diagnosis according to international guidelines
- No restrictions on gender, age, country and areas (e.g. urban, rural)

**Context**

**Intervention delivery**

**Delivery mechanisms**

- Individual/collective sessions
- Telephone counselling
- Interviews

**Delivery Agent**

- Pneumologist
- Primary care doctors
- Nurses/ therapists
- Pharmacists

**Setting**

- Inpatient and outpatient care
- Telephone-based setting
- Primary health practice
- Regional hospitals
- Community pharmacist

**Patient**

Attitudes, beliefs, knowledge, perceptions and expectations

**Healthcare professionals**

Involvement in education, Interaction and relation with patients, time dedication, prescriptions

**Society**

Socioeconomic status, family, friends
